# Marijuana and head and neck cancer: an epidemiological review

**DOI:** 10.1186/s40463-018-0319-2

**Published:** 2018-11-22

**Authors:** Michael Xie, Michael K. Gupta, Stuart D. Archibald, B. Stanley Jackson, James Edward Massey Young, Han Zhang

**Affiliations:** 1 Division of Otolaryngology—Head and Neck Surgery, McMaster University, G811, 50 Charlton Avenue E, Hamilton, ON L8N 4A6 Canada; 2 Michael G. DeGroote School of Medicine, McMaster University, Hamilton, ON Canada

**Keywords:** Marijuana, Head and neck cancer, Cancer epidemiology

## Abstract

**Background:**

Marijuana is the most widely used illicit substance in Canada. To date, no conclusive study has looked at the epidemiologic basis of marijuana use and head and neck cancer (HNC). Due to the imminent recreational legalization of marijuana in Canada, the epidemiologic relationship between marijuana use and HNC is becoming increasingly important.

**Objective:**

To examine the epidemiologic characteristics of HNC patients who are recreational marijuana users.

**Methods:**

This study was conducted at a single tertiary care centre from 2011 to 2014. Patients were enrolled consecutively at time of diagnosis of malignancy. Data was prospectively collected and included socioeconomic factors, alcohol/tobacco history, tumor characteristics, and treatment modality. Marijuana use was defined as current usage on an at least weekly basis.

**Results:**

Eight hundred seventy-nine patients met inclusion and exclusion criteria. Seventy-four (8.4%) patients were classified as marijuana users. Compared to non-users, marijuana users were less likely to be married (*p* = 0.048) and had less significant tobacco smoking history (*p* = 0.004). There were no significant differences between other socioeconomic factors or local and regional disease (*p* > 0.05). Marijuana users differed in the proportion of cancers stratified by primary site (*p* < 0.0001), with higher rates of p16+ oropharyngeal cancers, and treatment modality (*p* < 0.0001), with more use of chemoradiation.

**Conclusions:**

HNC patients who were marijuana users were less likely to be married and smoke tobacco. They have a distinct cancer site prevalence and are more likely to be treated by chemoradiation. Understanding the epidemiological breakdown of marijuana users amongst HNC patients will be a useful adjunct for future studies.

**Electronic supplementary material:**

The online version of this article (10.1186/s40463-018-0319-2) contains supplementary material, which is available to authorized users.

## Introduction

*Cannabis sativa*, otherwise known as marijuana, is the most commonly used illicit drug in Canada. The 2012 Canadian Community Health Survey-Mental Health found that 3.4 million (12.2%) Canadians aged 15 or older reported using marijuana in the past year and the prevalence of lifetime marijuana use was determined to be as high as 42.5% [1]. Canada’s trend towards recreational legalization has been providing impetus to better elucidate the association between marijuana and head and neck cancer (HNC). Regardless of the change in usage with recreational legalization, a sizable percentage of the Canadian population might have the potential to increase marijuana usage based on current statistics [1–3]. Despite this, there is a tangible gap within the current literature in understanding the epidemiological variations of HNC patients in Canada who are marijuana users compared to those who are not.

Bridging this gap in knowledge and understanding is important to better explore the relationship between marijuana and HNC. HNC patients, much like others who suffer from life altering oncology diagnoses, often report impacts on their quality of life secondary to psychiatric and physical symptoms in relation to diagnosis and treatment [4–8]. Marijuana has been proposed to have a potential therapeutic role in in these patients due to its ability to enhance relaxation, decrease stress, and improve quality of life; however there is a lack of direct evidence at this time [1, 9, 10]. Other than the potential mood effects of marijuana, its role as a carcinogen is not yet established as the literature has been inconclusive in finding a link between marijuana as a cause for HNCs [11–14]. Although specifics of such relationships between marijuana and HNC are beyond the scope of this current study, a better understanding of the epidemiology of marijuana use in HNC can assist future work in these areas of research.

It is therefore, the aim of this study to examine the epidemiological variations within patients who are marijuana smokers diagnosed with HNCs.

## Methods

This study received ethics approval from the Hamilton Integrated Research Ethics Board.

### Data collection

Consecutive patients were recruited prospectively and consecutively at the time of biopsy proven diagnosis of HNC from the Juravinski Cancer Center, Hamilton, Ontario from July 2011 to July 2014. All patient data was collected prospectively. Patients were included if they were greater than 17 years old and had a pathologically confirmed diagnosis of cancer of the head and neck. Patients were excluded if they had incomplete data sets.

Included patients completed a structured questionnaire (Additional file 1) that requested information on the following variables: marijuana use, education level, ethnicity, marital status, alcohol consumption, tobacco use, pack year history, income, employment status, age, gender, Karnofsky score. Diagnostic and treatment data were also collected and included: primary site of cancer, clinical T stage, clinical N stage, and modality of treatment. For oropharyngeal cancers, p16 status was also collected. Socioeconomic data was cross-referenced with Canada Census Data from years, 2011 and 2015. Cases were linked to income quintiles using patients’ postal codes.

### Statistical analysis

Patients were split into marijuana user and non-marijuana user groups based on a history of marijuana usage. Marijuana use was defined as current usage on an at least weekly basis. This is based on previous studies that have examined the association of marijuana use and HNC [15, 16]. All patients within the marijuana user group were using marijuana at the time of the study collection.

Descriptive statistics were used to compare patient demographics between marijuana smokers and non-smokers. Discrete variables were compared using Chi-squared or fischer’s exact probability tests. *P*-values less than 0.05 were considered statistically significant. All analyses were performed using SPSS 20.0 (SPSS Inc., Chicago, IL.).

## Results

Eight hundred seventy-nine total patients met inclusion and exclusion criteria. 74 (8.4%) patients in the study population were classified as marijuana users. 805 (91.6%) patients were classified as non-marijuana users.

Table 1 summarizes patient characteristics for the two groups. The marijuana group had a mean age of 62.26, which was not statistically significantly different to the mean age of 56.76 in the non-marijuana group (*p* = 0.068). There was a higher proportion of male patients within the marijuana group compared to the non-marijuana group, 85.1% vs 71.2% respectively (*p* = 0.011). The predominant ethnicity in both groups was Caucasian, 97.3 and 95.1% in the marijuana and non-marijuana groups, respectively (*p* = 0.537). Overall, marijuana and non-marijuana patients did not differ in alcohol use (*p* = 0.183), but marijuana patients trended towards heavier drinking with more patients reporting 5–20 and >  20 drinks per week (17.6% vs 11.0 and 18.9% vs 8.1%, respectively). Tobacco use also differed between the two groups. A smaller proportion of patients in the marijuana group had tobacco usage compared to the non-marijuana user group (27.0% vs 54.5%, *p* = 0.001). Additionally, fewer marijuana patients reported having a significant smoking history (greater than 21 pack-years) compared to the non-marijuana user group (21.6% vs 34.6%, *p* = 0.004). Marijuana users reported a marginally higher Karnofsky score (92.4% vs 87.0%) as compared to the non-marijuana user group, but this difference in quality of life was not statistically significant (*p* = 0.084).

**Table 1:** Patient Characteristics

Variable	Non-Marijuana	Marijuana	*p*-Value
N	805	74	
Mean Age	56.76	62.26	0.068
Gender			0.011
Male	573 (71.2)	63 (85.1)	
Female	232 (28.8)	11 (14.9)	
Ethnicity			0.537
Caucasian	766 (95.1)	72 (97.3)	
Black	7 (0.9)	1 (1.4)	
Asian/Indian Descent	19 (2.4)	0 (0)	
Latin Descent	8 (1.0)	1 (1.4)	
Native/Aboriginal	5 (0.6)	0 (0)	
Alcohol Use			0.183
None	279 (34.6)	23 (31.1)	
< 5 Drinks/Week	381 (47.3)	24 (32.4)	
5–20 Drinks/Week	89 (11.0)	13 (17.6)	
> 20 Drinks/Week	65 (8.1)	14 (18.9)	
Tobacco Use			0.001
Yes	439 (54.5)	20 (27.0)	
No	366 (45.5)	54 (73.0)	
Tobacco Pack Year History			0.004
< 20	526 (65.4)	58 (78.4)	
> 21	279 (34.6)	16 (21.6)	
Karnofsky Score	87.0	92.4	0.084

Socioeconomic characteristics are summarized in Table 2 and statistical analysis represents comparison of proportions between the groups. Patients in both groups reported similar levels of education with comparable amounts having at least a high school diploma or less (*p* = 0.984). Both groups’ level of income was equally distributed between each of the income quintiles (*p* = 0.744). There was no statistical significant difference in employment status between the two groups (*p* = 0.439); in both groups patients were predominantly retired or employed full-time. There was a statistically significant difference in marital status between the two groups with a lower proportion of patients within the marijuana group reported being married/common law (55.4% vs 63.2%; *p* = 0.048).

**Table 2:** Socioeconomic Characteristics

Variable	Non-Marijuana	Marijuana	*p*-Value
N	805	74	
Education Level, n (%)			0.984
Up to Grade 11	246 (30.6)	20 (27.0)	
High School Diploma	226 (28.1)	23 (31.1)	
Trade School	33 (4.1)	1 (1.4)	
College Diploma	146 (18.1)	19 (25.7)	
University Diploma	110 (13.7)	10 (13.5)	
Professional Degree	5 (0.6)	1 (1.4)	
Master’s Degree	25 (3.1)	0 (0)	
PhD	7 (0.9)	0 (0)	
Marital Status, n (%)			0.048
Married/Common Law	509 (63.2)	41 (55.4)	
Single	104 (12.9)	13 (17.6)	
Relationship	3 (0.4)	2 (2.7)	
Divorced	87 (10.8)	15 (20.3)	
Widowed	102 (12.7)	3 (4.1)	
Income Quintile, n (%)			0.744
1	159 (19.8)	15 (20.3)	
2	172 (21.4)	15 (20.3)	
3	163 (20.2)	15 (20.3)	
4	163 (20.2)	15 (20.3)	
5	148 (18.4)	14 (19.0)	
Employment Status, n (%)			0.439
Full Time	288 (35.8)	25 (33.8)	
Part Time	27 (3.3)	1 (1.4)	
Retired	399 (49.6)	35 (47.3)	
Unemployed	40 (5.0)	3 (4.1)	
Disability	44 (5.5)	10 (13.5)	

Tumor characteristics are summarized in Table 3 and statistical analysis represents comparison of proportions between the groups. The distribution of primary cancer sites was significantly different between marijuana users and the non-marijuana user group (*p* < 0.0001). There was a statistically significantly higher amount of oropharynx cancer within the marijuana user group (63.5% vs 19.9%, *p* < 0.0001). As well, a higher percentage of the marijuana group’s oropharyngeal cancers were found to be p16 positive (95.7% vs 82.5%, *p* = 0.002). Although the overall distribution of primary cancer sites were statistically different (*p* < 0.0001), similar rates of malignancy were observed between the marijuana and non-marijuana groups at the oral cavity (18.9% vs 17.5%), hypopharynx (1.4% vs 1.5%), and larynx (16.2% vs 17.2%). Even though no statistically significant differences were observed for the cT stage (*p* = 0.076) and cN stage (*p* = 0.144), treatment modality differed significantly between the two groups (*p* < 0.0001). Marijuana patients were more likely to receive chemoradiation (48.6% vs 24.2%) and less likely to receive surgery alone (10.8% vs 20.8%) as compared to non-marijuana users (*p* < 0.0001).

**Table 3:** Tumor Characteristics

Variable	Non-Marijuana	Marijuana	*p*-Value
N	805	74	
Primary Site of Cancer, n (%)			< 0.0001
Oropharynx	160 (19.9)	47 (63.5)	
Oral Cavity	141 (17.5)	14 (18.9)	
Nasopharynx	19 (2.4)	0 (0)	
Hypopharynx	12 (1.5)	1 (1.4)	
Larynx	138 (17.2)	12 (16.2)	
Skin	81 (10.1)	0 (0)	
Salivary Gland	76 (9.4)	0 (0)	
Other	177 (22.0)	0 (0)	
Oropharynx p16 Status			0.002
P16 Positive	132 (82.5)	45 (95.7)	
P16 Negative	28 (17.5)	2 (4.3)	
cT-Stage, n (%)			0.076
Tis	13 (1.6)	1 (1.4)	
T1	245 (30.4)	22 (29.8)	
T2	318 (39.5)	28 (37.8)	
T3	148 (18.4)	14 (18.9)	
T4	81 (10.1)	7 (9.6)	
cN-Stage, n (%)			0.144
N0	357 (44.4)	31 (43.0)	
N1	100 (12.4)	8 (11.1)	
N2a	229 (28.4)	20 (27.8)	
N2b	59 (7.4)	6 (8.3)	
N2c	30 (3.7)	3 (4.2)	
N3	30 (3.7)	4 (5.6)	
Treatment, n (%)			< 0.0001
S	167 (20.8)	8 (10.8)	
RT	151 (18.8)	14 (18.9)	
CRT	195 (24.2)	33 (48.6)	
S-RT	197 (24.4)	12 (16.2)	
S-CRT	84 (10.4)	6 (8.1)	
Palliative	11 (1.4)	0 (0)	

## Discussion

The first discussion about the demographics of marijuana use in HNC patients was by Donald in 1986 [17]. In a case series of six advanced head and neck squamous cell carcinoma cases, which were regular marijuana users, he commented on the exceptionally young age of the group, at a mean age of 27.1, as well as the fact that 2 out of 6 patients had never smoked tobacco [17]. This case series was the first to hint at a possible epidemiological variant in marijuana smoking HNCs. Since then case-control studies have examined the demographics of their respective cases and controls, although they have presented limited specific epidemiological data on the marijuana user cases.

The INHANCE pooled analysis combined five case control studies with 4029 HNC cases from sites in United States and South America, but did not include data from Canada [18]. The prevalence of marijuana use was found to be 10.1% amongst cases and there was a high proportion of males at 85.2%, comparable to the 8.4% prevalence and 85.1% male percentage observed in our study. Marijuana users also similarly reported less tobacco and alcohol use compared to controls. The INHANCE study also found cancers at the oropharyngeal subsite most associated with marijuana, reflective of the subsite distribution observed in our data [18]. The population was more ethnically diverse compared to our study group with only 18.3% of HNC cases being Caucasian, yet similar trends were observed [18]. A follow-up INHANCE analysis of an oropharyngeal subsite reiterated the relationship of this subsite with marijuana which increased with frequency and duration of use. Unfortunately, demographic and socioeconomic data was not compared between the marijuana users and non-users in the group. Marijuana’s relationship with the oropharynx subsite was similarly found to be marginally associate with low smoking/alcohol use but potentially confounded by HPV exposure [16]. However, the primary goal of the INHANCE analyses were not to examine epidemiological variation and only limited comparisons can be made to our data with regards to p16 status, marriage, income, employment, clinical stage, and treatment regime as they were not examined.

Based on 2012 Canada census data, there are a few demographic patterns seen in marijuana users that are part of the general Canadian population [1]. They tend to be younger with 33% being 18–24 and only 0.8% aged 65 or older [1]. There is a male predominance in consistent users with 4.6% of males vs 1.7% of females reporting weekly use and 2.4% of males and 1.2% of females reporting daily use [1]. Lifetime marijuana use and marijuana use within the past year were also higher in males compared to females at 49.4% vs 35.8 and 16.1% vs 8.3%, respectively [1]. In contrast to the young age of marijuana smokers in the general population, our study’s marijuana users were older with a mean age of 62.26. Based on at least weekly marijuana usage, our study observed a 5.7:1 male predominance in the marijuana user group; this is higher than the male predominance observed in the general population both in weekly users (3:1) and daily users (2:1) [1]. The differences in gender and mean age between our population subset compared to that of Canada census data could be related to the epidemiology of HNC where the mean age often range from 55 to 65 years old depending on the disease site and are often predominantly male [19]. While Canada census data provides an overview of the general population, our cross sectional sample of HNC cannot be discounted.

The epidemiology of HNCs related to tobacco smoke and alcohol have also been well studied. Patients have been shown to have peak incidence in late-middle age at 55–59 with a significant 5:1 male predominance [20, 21]. The proportion of patients having an educational attainment higher than a high school diploma are similar between the tobacco and alcohol related HNCs at 29.2 and 31%, respectively [20, 21]. This is similar to patients within our non-marijuana user population where the mean age was 56.6 with a predominantly male population. Our study’s marijuana group however, presented slightly later in late-middle age at 62.26 but with a similar 5.7:1 male predominance. Moreover, the marijuana group had higher educational attainment with 41.9% of patients having higher than a high school diploma. It is still unclear whether these subtle demographic variations within the marijuana user HNC population have any significant effect on the treatment or survivorship outcomes. Certainly more knowledge on this subgroup of patients is needed in the future and could provide interesting insights.

The only variation in socioeconomic characteristics identified from our data set was that HNC patients that reported marijuana use are less likely to be married/common law compared to those that did not (55.4% vs 63.2%; *p* = 0.048). Marital status has been shown to be an independent prognostic factor in HNCs; however, given the other differences in this population, the significance or impact of marital status among marijuana smoking HNC patients remains undetermined [22]. It is interesting that the marijuana user group were not only more likely to be single but also had higher rates of HPV positive oropharynx cancer, a factor associated with increased sexual practices and partners [15]. While quantification of the amount of partners within this subsite group of patients was beyond the scope of this study future studies is warranted to delineate any potential relationships. The remaining socioeconomic characteristics including educational attainment, income, and employment status were not different between the HNC patients in the marijuana and non-marijuana groups.

HPV positive oropharyngeal cancer was the site of the highest prevalence in the marijuana user group compared to the non-user group (oropharyngeal cancer 63.5% vs 19.9%, *p* < 0.0001; p16 positive 95.7% vs 82.5%; *p* = 0.002). This is reflective of the shift to HPV positive oropharynx cancers as the predominant head and neck disease site in Ontario and North America [23–25]. The difference in treatment modality between the two groups supports this as the marijuana user group had statistically higher chemoradiation (*p* < 0.0001) as the primary therapy, which is the standard treatment option for this disease site within our cancer center. Interestingly, patients who were marijuana users were also found to have statistically lower incidence of tobacco use (*p* = 0.001). This coupled with the higher incidence of HPV positive oropharynx cancer within the marijuana user group suggest that patients with recreational marijuana use are reflective of the trends in epidemiological variation of HNC patients. These variations could suggest differences in sexual practices between marijuana users and non-users, the potential direct oncogenic effects of marijuana use, or something entirely different that is not yet defined. While the establishment of a true cause and effect relationship is beyond the scope of this study, it is intriguing and would certainly warrant further research.

This is a population-based study with prospectively collected data but is subject to limitations. The primary limitation of this study is the small sample size of HNC patients that reported marijuana use. We were also unable to sub-stratify the marijuana user group based on the quantity of use. Unlike smoking and alcohol, marijuana has not been concretely established as a risk factor for HNC and there is no validated clinically significant cut-off for marijuana frequency/use. We elected to use the definition of at least weekly use based on extrapolation of findings in the literature. Within the setting of HNC, there is data to show marijuana use at a frequency of less than three times per week or at least once monthly is associated with oropharyngeal and HPV-related cancers [15, 16]. Beyond oncology, at least weekly cannabis use has been found to be predictive of adverse events in the context of psychosis, neuropsychological function, and stroke/TIA [26–29]. In addition, since cannabis remains classified as Schedule II substance under the Canadian Controlled Drugs and Substances Act, there may be an under-reporting of marijuana use in the study population which may affect the subsequent break down of patient as well as socioeconomic characteristics of patients [30]. Patient income quintiles were extrapolated from neighbourhood level income and thus the interpretation may be susceptible to ecological fallacy. Despite potential discordance, neighbourhood and individual level income have been shown to produce comparable observations [31, 32]. Due to the inadequate follow-up data, we also were not able to discover the potential effects of marijuana on HNC patients. Future long-t erm prospectively based studies would help mitigate and answer more questions on the relationship of marijuana on HNC patients.

To our knowledge this is the first study to look at the epidemiological variances within HNC patients who are marijuana users. Patients were found to have predominantly HPV positive oropharynx cancer and more likely to be single with statistically significant less tobacco use. There was no statistically significant difference between the two groups in cT and cN Stage as well as age at diagnosis, alcohol use, Karnofsky score, education level, ethnicity, employment status, and income quintiles. This study has also highlighted variations in epidemiology compared to marijuana users in general and HNC patients that smoke and use alcohol.

## Conclusion

Patients diagnosed with HNC who are marijuana users do present with subtle variation in tumor and socioeconomic characteristics when compared with non-marijuana users. This study provides an overview of these epidemiological variations and will be a useful adjunct for future studies within this area that explore the potential oncologic as well as quality of life of life effects marijuana has on HNC patients.

## Additional file

Additional file 1: Juravinski Cancer Center Head and Neck Cancer Intake Database. (DOCX 15 kb)
