# Supplementary material for: Marijuana and head and neck cancer: an epidemiological review
Source: J Otolaryngol Head Neck Surg. 2018 Nov 22;47:73. doi: 10.1186/s40463-018-0319-2 (PMC6249858; doi:10.1186/s40463-018-0319-2)
Supplement: Supplementary file 1 — Juravinski Cancer Center Head and Neck Cancer Intake Database. (DOCX 15 kb) [file 40463_2018_319_MOESM1_ESM.docx]

Appendix I

Hamilton Region Head and Neck Cancer Database

DATA COLLECTION FORM

1. Patient’s full name __________________________________________________________________
2. Medical record number ______________________________________________________________
3. Date of birth (mm/dd/yyyy) ___________________________________________________________
4. First three digits of postal code ________________________________________________________
5. Date first seen in head and neck clinic (mm/dd/yyyy) ______________________________________
6. Head and neck clinic appointment site (JCC, SJH) __________________________________________
7. Diagnosis _________________________________________________________________________
8. Demographics and additional patient characteristics and risk factors
   1. Sex ________________________________________________________________________
   2. Race _______________________________________________________________________
   3. Marital status _______________________________________________________________
   4. Education level ______________________________________________________________
   5. Employment ________________________________________________________________
   6. Income _____________________________________________________________________
   7. Alcohol use (units per week) ____________________________________________________
   8. Inhalation tobacco use (quit, and if so how long ago? Pack years, current packs/cigarettes/day) _________________________________________________________
   9. Second hand smoke exposure (Yes/No) ___________________________________________
   10. Chewing tobacco use (quantity per day/week/length if use; quit?) ___________________________________________________________________________
   11. Smoked marijuana use (current/previous, frequency of use) ___________________________________________________________________________
   12. Regularly see a dentist (Yes/No, check-ups per year) ________________________________
   13. Environmental exposure
       1. Arsenic ______________________________________________________________
       2. Sun (and skin type) _____________________________________________________
       3. Heavy metal exposure (cadmium and nickel) ________________________________
       4. Wood dust exposure ___________________________________________________
       5. Asbestos _____________________________________________________________
       6. Radiation exposure (environmental or radiation for cancer treatment in past) ____________________________________________________________________
       7. Betel nut exposure _____________________________________________________
   14. Immunocompromised state ____________________________________________________
9. Tumor data ________________________________________________________________________
   1. Date of first diagnosis (mm/dd/yyyy) _____________________________________________
   2. Type of tumor (primary, secondary, recurrent) _____________________________________
   3. Tumor site __________________________________________________________________
   4. Tumor subsite _______________________________________________________________
   5. Side specification (laterality) ____________________________________________________
   6. Histology (immunohistochemistry stains (e.g. p16, p63) and grade _____________________
   7. cTNM ______________________________________________________________________
   8. pTNM (if applicable) __________________________________________________________
   9. Functional status (Karnofsky score) ______________________________________________
10. Comorbidities

______________________________________________________________________________________________________________________________________________________________________________________________________________________________________________________

1. Treatment Data
   1. Therapy intent _______________________________________________________________
   2. Treatment modality __________________________________________________________
   3. Surgery
      1. Date of initial surgery (mm/dd/yyyy) ______________________________________
      2. Type of surgery _______________________________________________________
      3. Neck dissection _______________________________________________________
      4. Reconstruction ________________________________________________________
   4. Chemotherapy
      1. Adjuvant, neoadjuvant, concomitant ______________________________________
      2. Date of initial chemotherapy (mm/dd/yyyy) _________________________________
      3. Chemotherapy ________________________________________________________
   5. Radiotherapy
      1. Adjuvant, neoadjuvant, concomitant ______________________________________
      2. Radiation therapy technique _____________________________________________
      3. Fractionation scheme (total dose, fraction dose, delay or disruption of treatment) ____________________________________________________________________
      4. Date of first treatment (mm/dd/yyyy)______________________________________
      5. Residual status _______________________________________________________
      6. Documentation of treatment success ______________________________________
      7. End of treatment date (mm/dd/yyyy)______________________________________
      8. Status of disease (complete response, partial response, progressive disease, stable disease) _____________________________________________________________
2. Inventory data
   1. The Head and Neck Symptoms Scale of the University of Washington Quality of Life Questionnaire (UNHNSS)
   2. Edmonton Symptom Assessment Scale (ESAS)
   3. EuroQOL 5D (EQ-5D)
